# Ultrahigh Energy and Power Densities of d-MXene-Based Symmetric Supercapacitors

**DOI:** 10.3390/nano12193294

**Published:** 2022-09-22

**Authors:** Beenish Mustafa, Wengang Lu, Zhiyuan Wang, Fuzhuo Lian, Andy Shen, Bing Yang, Jun Yuan, Chang Wu, Yangbowen Liu, Weiwei Hu, Lei Wang, Geliang Yu

**Affiliations:** 1 National Laboratory of Solid-State Microstructures, School of Physics, Nanjing University, No. 22, Hankou Road, Nanjing 210093, China; 2 Hubei Jiufengshan Laboratory, Wuhan 430206, China; 3 Jiangsu Industrial Technology Research Institute, Nanjing 210093, China; 4 Collaborative Innovation Centre of Advanced Microstructures, Nanjing University, Nanjing 210093, China

**Keywords:** symmetric supercapacitors, MXene, chloroauric acid (HauCl_4_), energy density, power density, aqueous electrolyte, energy storage, 2D materials

## Abstract

Here, rational design electrodes are fabricated by mixing MXene with an aqueous solution of chloroauric acid (HAuCl_4_). In order to prevent MXene from self-restacking, the groups of -OH on the surface of Ti_3_C_2_T_x_ nanosheets underwent a one-step simultaneous self-reduction from AuCl_4_-, generating spaces for rapid ion transit. Additionally, by using this procedure, MXene’s surface oxidation can be decreased while preserving its physio-chemical properties. The interlayered MX/Au NPs that have been obtained are combined into a conducting network structure that offers more active electrochemical sites and improved mass transfer at the electrode–electrolyte interface, both of which promote quick electron transfer during electrochemical reactions and excellent structural durability. The Ti_3_C_2_T_x_-AuNPs film thus demonstrated a rate performance that was preferable to that of pure Ti_3_C_2_T_x_ film. According to the results of the characterization, the AuNPs effectively adorn the MXene nanosheets. Due to the renowned pseudocapacitance charge storage mechanism, MXene-based electrode materials also work well as supercapacitors in sulfuric acid, which is why MXene AuNPs electrodes have been tested in 3 M and 1 M H_2_SO_4_. The symmetric supercapacitors made of MXene and AuNPs have shown exceptional specific capacitance of 696.67 Fg^−1^ at 5 mVs^−1^ in 3 M H_2_SO_4_ electrolyte, and they can sustain 90% of their original capacitance for 5000 cycles. The highest energy and power density of this device, which operates within a 1.2 V potential window, are 138.4 Wh kg^−1^ and 2076 W kg^−1^, respectively. These findings offer a productive method for creating high-performance metal oxide-based symmetric capacitors and a straightforward, workable approach for improving MXene-based electrode designs, which can be applied to other electro-chemical systems that are ion transport-restricted, such as metal ion batteries and catalysis.

## 1. Introduction

In order to use sustainable energy sources such as solar, wind, geothermal, tidal, or biomass, many energy conversion and storage technologies have been created so far, including the solar cell, flywheel, compressed air, fuel cell, supercapacitor, and battery [1,2,3]. Supercapacitors (SCs), which have higher power and energy density than batteries, are widely considered to be the energy storage technology of the future [4,5].

Due to the numerous wearable and portable electronics applications of energy storage technologies such as SCs, these devices are fascinating. However, due to the rigidity of their constituent parts, classic SCs are often stiff [6,7]. Additionally, because of their large electrochemical active area, 2D solids have sparked the most attention among the various electrode materials available for supercapacitors [8].Typical porous carbon (PC)- based electrodes additionally require a conducting substance (such as carbon black), a current collector (such as nickel foam), and an inactive binder (such as PTFE and PVDF). As a result, the weight and volume of the SCs are inevitably increased, which severely limits their applications. Furthermore, the adverse effects of these chemicals during the charging-discharging process include plugging PC pores, lowering electrode conductivities, and decreasing active surface area [9,10]. In order to develop high-performance SCs with flexibility and ultra-thinness, it is essential to create a freestanding electrode that combines great conductivity, mechanical qualities, as well as great electrochemical activity [11,12,13].

MXene, a class of two-dimensional substances, is frequently used as a supercapacitor electrode material [14,15,16,17]. Typically, ternary-layered carbides of MAX phases are used to create MXene by etching off the A-layer [18]. The typical formula for this compound is M_n+1_ X_n_T_x_, where M is a transition metal, A is an element from group 13 or group 14 of the periodic table, X is carbon or nitrogen, and T_x_ is a surface functional group [19]. Rich surface functional groups, high metal conductivity, good hydrophilicity, and a sizable specific surface area are among the properties of MXene [20,21,22]. The large specific area surface of MXene’s 2D sheets guarantees a high electric double-layer capacitance. However, more critically, the redox reactions of its abundant surface terminations, especially in acidic conditions, supply MXene sheets with additional pseudocapacitance [4,23]. MXene (Ti_3_C_2_T_x_) has recently received a lot of interest in energy storage, notably supercapacitors, because of its unusual physical and chemical properties [24,25,26].

MXene has a substantial specific surface area in a planar geometry. MXene makes it simple to create thin films with great mechanical properties and excellent flexibility due to the few atomic layers and a less wrinkly flat surface [11,12,13]. More importantly, because of their superior volumetric capacitance, flexibility, and mechanical stability, the free-standing MXene films can be used immediately for flexible electrodes after vacuum filtering [18,27,28]. Aggregation and restacking of MXene nanosheets during electrode fabrication, like other 2D nanomaterials, seriously hinders the rapid diffusion of electrolyte ions and affects the utilization of the active surface of the electrodes to its full potential, resulting in a limited specific volumetric capacitance, especially at higher scan rates [16,29]. As a result, high-performance electrodes must be developed in order to couple more ideal candidate materials with MXene to ensure that the potentials of both materials are expressed. To address these issues and to prevent stacking while increasing ion transport and electrolyte penetration, various interlayer spacers are used in 2D materials. These include RGO/AuNPs [30], MXene/CNTs [31], MX/MoO_3_ [32], MX/MoS_2_ [33], 400-KOH-Ti_3_C_2_ MXene [4], MX/NF/PC [27] and EDA-Ti_3_C_2_T_x_ [34]. Previous studies have shown that when AuNPs are used with graphene [35,36,37,38,39,40,41], they have shown excellent results that prevent stacking, and speed up ion diffusion and electrolyte penetration, so they can be used in energy storage devices [42].

The conductive, flexible MXene/Au membrane was fabricated by liquid phase exfoliation followed by a vacuum filtration process. The pre-made MXene and chloroauric acid (HauCl_4_) solution were mixed well with the help of a magnetic stirrer and filtered with vacuum filtration.

As Ti_3_C_2_T_x_ possesses outstanding metallic conductivity, it can form films and is a good binder for point-to-point electrical contact between two molecules, while Au inclusion inhibits Ti_3_C_2_T_x_ self-restacking. Resultantly, the prepared MX/Au film may have significant interlayer spacing and high flexibility. Therefore, this study shows, for the first time, the integration of solution-processable MX/Au films in 3 M H_2_SO_4_ as a flexible self-supporting electrode for symmetric SCs. In this work, a simple and practical way to develop enhanced MXene-based free-standing, flexible electrodes with exceptional electrochemical versatility and performance were chosen, which are promising for future use in portable electronics. This research advanced our understanding of intercalating noble metal and nanocomposite electrodes to improve MXene’s electrochemical performance, proposing its new applications. The proposed supercapacitor delivers a high specific capacitance, outperforming the vast number of MXene-based symmetric SCs. Furthermore, the composite film presents high deformation, thanks to its ultrathin construction, and can easily be folded, bent, and twisted. This study offers a straightforward and practical method for creating improved MXene-based free-standing, flexible electrodes with excellent electrochemical versatility and performance, demonstrating tremendous promise for use in portable electronics of the future.

## 2. Experimental Procedure

### 2.1. Materials

Layered ternary carbide powders (Ti_3_AlC_2_), lithium fluoride (LiF), sulfuric acid (H_2_SO_4_), and hydrochloric acid (HCl, 35–38%) were purchased from Rhawn Co., Ltd., Shanghai, China. HAuCl_4_ was purchased from Chemical Energy Reagent Co., Ltd., Shanghai, China. Nylon membrane was bought from Whatman, and cellulose membrane from Shanghai Xingya company, Shanghai, China.

### 2.2. Preparation of Delaminated MXene Nanosheets

In a nutshell, a 20 mL solution of 12 M HCl was combined with 1.6 g of LiF (Aladdin, 99 percent) and agitated for 25 min at 40 °C using a magnetic Teflon stir bar. The reaction was then maintained for 24 h at 40 °C to etch the aluminum atoms in the Ti_3_AlC_2_ phase as Ti_3_AlC_2_ powder, 1 g, was added gradually to the aforementioned solution for the following 20 min. The mixture was then rinsed at least seven times with DI water until the supernatant pH was 7. Following the collection of the delaminated sediment and preparation of the MXene suspension (d-Ti_3_C_2_T_x_), 40 mL of deionized water was added, and the mixture was further treated ultrasonically, pulsating for 2 s at a 35% intensity for 2 h while being kept at 5 °C in an ice bath. After being collected and centrifuged for 1 h at 3500 rpm, the supernatant obtained had a greenish-black color, and the sediment was decanted away. Notably, the amount of suspension at a specific concentration allows for exact control of membrane thickness. The precise concentration of the suspension was ascertained by weighing the filtered membranes after vacuum filtering a specific amount of the MXene suspension.

### 2.3. Preparation of MXene/AuNPs Composite

The MXene and AuNPs composites were prepared by taking 0.36 g of HAuCl_4_ powder with 47.8% Au content and dissolving it into 36 mL of DI water. When it was fully dissolved, only 0.3 mL of solution was added to the 15 mL of MXene solution. The corresponding mixture was named MX/Au. After 30 min of magnetic stirring at 350 rpm, the final solution was filtered through a 0.2 µm Whatman nylon membrane filter and dried at room temperature. Finally, flexible and freestanding MXene/AuNPs composite film was obtained after peeling it off from the filter paper, punched into the desired shape, and used for electrodes without any further modification.

### 2.4. Characterization of Materials

The microstructure and morphology of delaminated MXene and MX/Au free-standing electrodes were investigated by environmental scanning electron microscope ESEM (Quanta200, FEI, USA) adjusting at high vacuum mode, 3.0 nm spot, voltage ~15–20 KV coupled with energy dispersive X-ray spectroscopy EDX (Ulti Max 100, Oxford, UK) at spot 3.0 with a voltage of 10 KV. In addition, a high-resolution transmission electron microscope (HRTEM) (Tecnai F20, FRI, Ames, IA, USA) equipped with a field-emission gun at an accelerating voltage of 200 kV was used to examine the micromorphology of the films in more detail using selective area electron diffraction (SAED). ImageJ was used to compute the distance between the lattice fringes. The crystallographic and phase examinations were carried out using X-ray diffraction (XRD, X’TRA) equipment with Cu K1 radiation (d = 0.154 nm) and a step size of 0.02° from 3° to 80°. For compositional analysis, XPS (PHI5000VersaProbe, ULVAC-PHI, Chigasaki, Japan) was used for X-ray photoelectron spectroscopy (XPS).

### 2.5. Electrochemical Evaluation

To investigate the capacitance capabilities of the SCs, all electrochemical measurements were conducted on the electrochemical workstation CHI660E using a two-electrode Swagelok setup. The three-electrode characterization was also performed in which Ag/AgCl was used as the reference electrode and a platinum sheet electrode as the counter electrode. To identify the voltage window and comprehend the capacitive behaviors from 5 mVs^−1^ to 100 mVs^−1^, cyclic voltammetry (CV) was used. The specific capacitances were assessed using galvanostatic charge–discharge measurement (GCD). In a frequency range of 10 MHz to 100 kHz, electrochemical impedance spectroscopy (EIS) was conducted at an open circuit voltage and an amplitude of 5 mV. The CV test was run for 5000 cycles at a scan rate of 50 and 100 mVs^−1^ to assess long-term cycling stability. Before any measurements, each electrode was cycled at least 50 times at a scan rate of 20 mVs^−1^ in a series of pre-experiments to choose an appropriate potential range and ensure the electrochemical behavior was not caused by the electrolyte degrading. It was decided to select the potential range until there was no discernible gas evolution. Equations were used to figure out the electrode materials’ specific capacitances and the power and energy densities of the symmetric SCs.

## 3. Results and Discussion

### 3.1. Sample Preparation and Surface Studies

**Figure 1:**
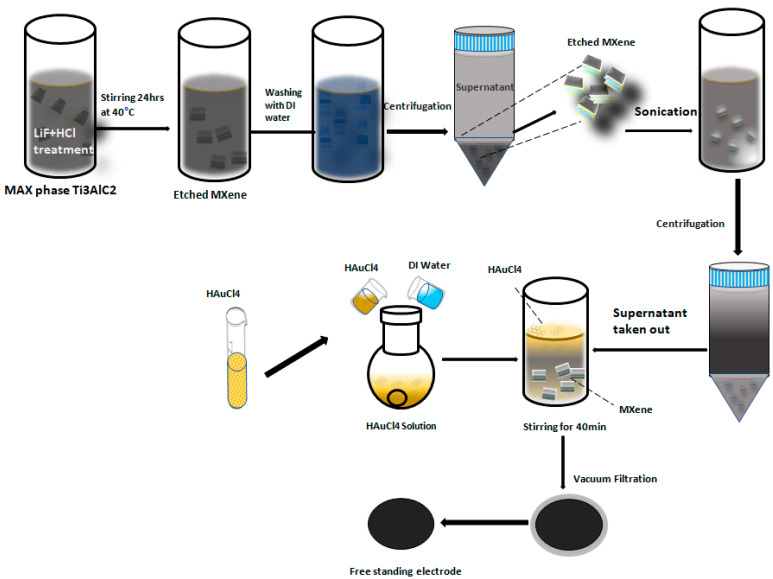
Schematic Illustration of the fabrication of MX/AuNPs free-standing film.

Figure 1 depicts a schematic illustration of the manufacturing of MX/Au films. A few-layered Ti_3_C_2_T_x_ MXene nanosheet colloidal suspension was prepared by selectively etching a Ti_3_AlC_2_ in a LiF and HCl solution, followed by centrifugation, ultrasonication, and vacuum filtration. Simple mixing and stirring allowed the HAuCl_4_ molecules to intercalate into the Ti_3_C_2_T_x_ layers. As the HAuCl_4_ aqueous solution is highly reducible, no other reducing agent was used. Thus, the self-supporting MXene-based 2D MX/Au film was successfully obtained after simple vacuum filtering (Figure S2b). Additionally, the membrane was very flexible as it wrapped around without losing integrity (Figure S2a).

**Figure 2:**
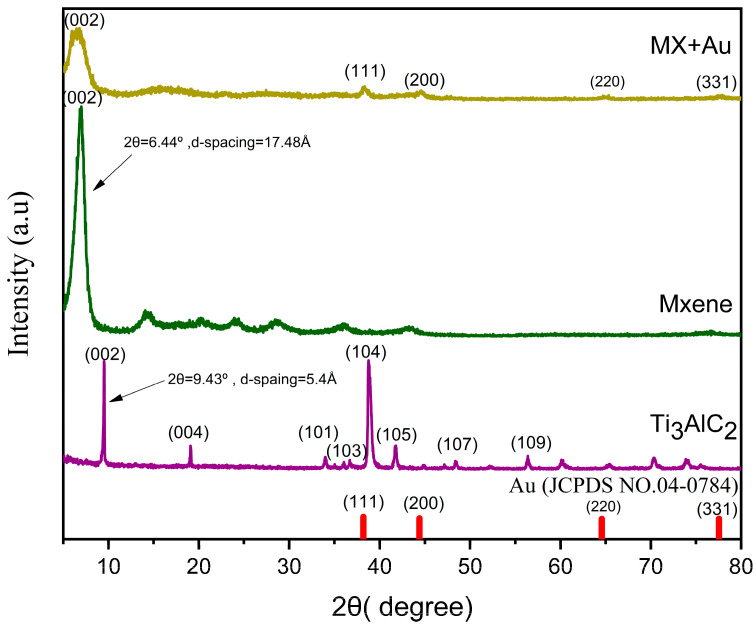
XRD patterns of Ti_3_AlC_2_, Ti_3_C_2_T_x_, AuNPs, and MXene/AuNPs.

The results of XRD characterization of Ti_3_AlC_2_, Ti_3_C_2_T_x_, and MX/Au are presented in Figure 2. The characteristic peaks of Ti_3_AlC_2_ at 9.43° with d-spacing of 5.4° and 38.82° are at (002) and (104) locations of crystal planes. After the etching and delamination procedure, the strong peak (104) vanished, indicating that the aluminum (Al) atomic layer of Ti_3_AlC_2_ was successfully etched. The angle of the MXene peak (002) is 6.44°, which is lower than that of Ti_3_AlC_2_ at 9.43°, demonstrating that, following the etching and delamination process, the space between layers has been increased. The sharpness of the (002) peak, which stays prominent, indicates that the generated MXene has a higher degree of crystallinity and a well-ordered structure. The (002) peak location of MXene in MX/Au film remained unaltered, showing that MXene exists, but there is no gold incorporated into the MXene lattice [43]. Further, nanocomposite samples displayed the characteristic peaks at 2θ = 38.2°, 44.4°, 64.6°, 77.5°, and 81.7°, which are attributed to crystal planes of (111), (200), (220), (222), and (331) face-centered cubic (fcc) AuNPs (JCPDS no. 04–0784) [44]. The nanocomposite samples of XRD patterns demonstrate that AuNPs were successfully prepared.

**Figure 3:**
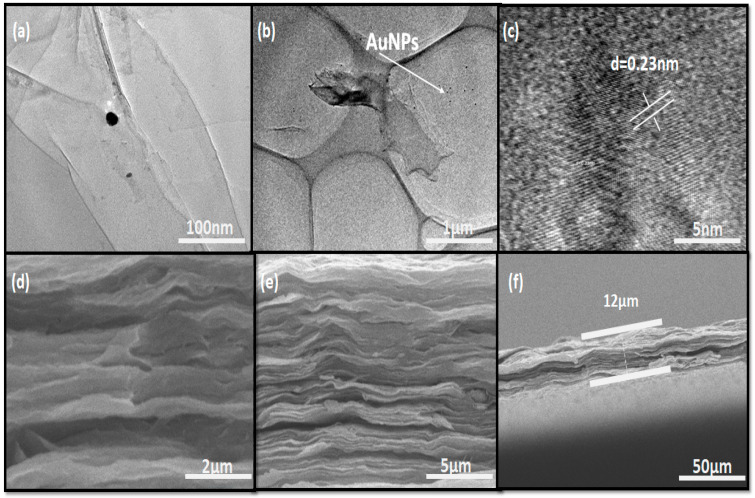
(**a**,**b**) TEM image of MX/AuNps; (**c**) HRTEM image of MX/AuNPs; (**d**) SEM images of MX/AuNPs composite film; (**e**) cross-sectional image of the MX/AuNPs composite film demonstrating stacked layered structure; (**f**) cross-sectional image showing the thickness of the composite film.

Following material characterization, environmental scanning electron microscopy was used to examine the morphology and microstructure of several pure MXene films and MX/Au. The SEM pictures of the top and side views of the MXene and MX/Au are shown in Figure 3d–f and Figure S5a,b. The side view displays MX/Au stacked layers, and the cross-sectional image is shown in Figure 3d,e. The top view of the film in Figure S5a clearly demonstrates that the surface of the film is wrinkled. The morphologies of MX/Au are comparable to those in pure MXene film (Figure S5a,b), but the surface is rougher. The film has layers of crumpled shape as shown in Figure 3d. The cross-sectional SEM images of MX/Au hybrid films (Figure 3e,f) show that the lamellar structure is well-aligned. So, it has been shown that AuNps can solve the self-stacking problem of Ti_3_C_2_T_x_ nanosheets, increasing their active surface for energy storage. In the meantime, MXene could work as a binder to prevent the loss of active components during the charging and discharging process. The EDS spectrum and elemental mapping of MXene film has been shown in Figure S4a,b respectively. Additionally, the MX/Au hybrid film displays the characteristic peaks of MXene and AuNPs (Figure 2), demonstrating that the coexistence of AuNPs does not affect the stacking order of Ti_3_C_2_T_x_ nanosheets. By using elemental mapping investigations, which clearly demonstrate the homogenous distributions of Ti, C, O, and Au elements inside MX/Au film, the compositional distributions of the MX/AuNps composite film (Figure S3a,b) were confirmed. Table S1 shows the elemental values of MXene and MX/AuNps composites. Additionally, one of the significant evaluation standards for flexible energy storage systems is the flexibility of the electrodes. As shown in Figure S2a, the MX/Au hybrid film demonstrates outstanding flexibility and high deformation, as it can be bent around a glass rod and folded repeatedly without cracking. After being treated with HAuCl_4_ with no other reducing agents, Ti_3_C_2_T_x_ nanosheets are adorned with AuNPs, as shown by the EDS of MXene/Au in Table S1. The HR-TEM image (Figure 3c) shows the d-spacing of 0.23 nm, which is in accordance with the (111) interlayer spacing of Au. Figure 3a,b of TEM clearly shows AuNPs on Ti_3_C_2_T_x_ nanosheets. The TEM and HRTEM images of MXene nanosheets have been shown in Figure S5c,d.

**Figure 4:**
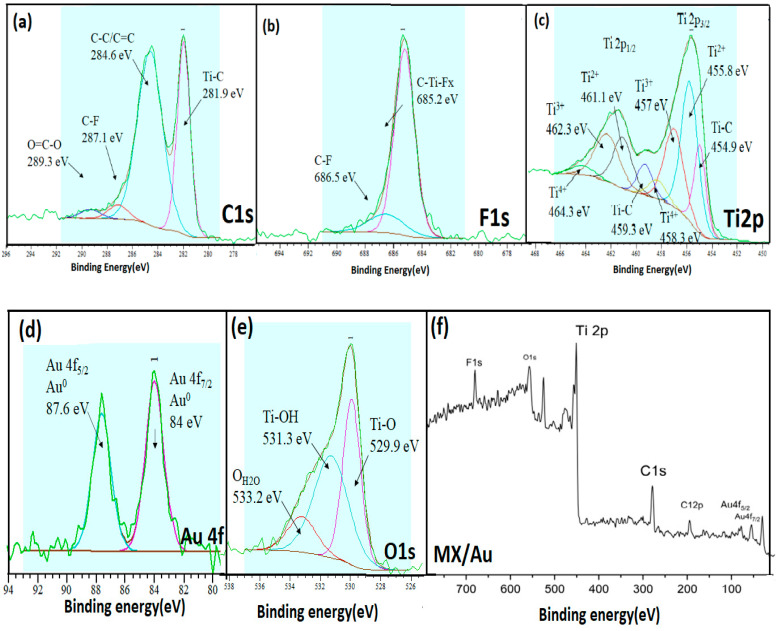
High resolution XPS spectra of (**a**) C 1s of MX/AuNPs; (**b**) F 1s; (**c**) Ti2p; (**d**) Au 4f; (**e**) O 1s; (**f**) survey spectra of MXene/AuNPs film.

The chemical bonding states, as well as elemental compositions of fabricating electrodes, were also examined by using XPS. Figure 4 displays the complete XPS spectra of MXene and MX/Au. Figure 4f and Figure S9 show the survey spectrum of MX/AuNPs and MXene. The common peaks of Ti 2p, C 1s, F 1s, and O 1s from 0 to 800 eV demonstrate the presence of MXene and -F, -Cl, -OH, and -O groups. The C 1s spectra of MX/Au contain distinct peaks at 289.3, 287.1, 284.6, and 281.9 eV, which correspond to the groups of O=C-O, C-F, C-C/C=C, and Ti-C, as shown in Figure 4a. The Ti 2p spectra of MX/Au are indexed with four characteristic peaks located at 454.9, 455.8, and 457, 458.3 eV, which are comparable to tetravalent Ti-C, Ti-X (TiCx, x < 1), and TixO, respectively, as shown in Figure 4c. The O 1s spectra of MX/Au contain four distinctive peaks located at 529.9, 531.3, and 533.2eV, which correspond to the groups of absorbed O, Ti-O, and Ti-OH, respectively (Figure 4e). The F1s spectra of MX/Au are indexed with two peaks, 685.2 eV and 686.5 eV, as shown in Figure 4b. The Au 4f spectrum of MX/Au can be seen in Figure 4d to have two distinct peaks at 84.0 and 87.6 eV with a 3.6 eV binding energy difference, indicating the presence of Au. The aforementioned findings show that AuNPs are equally enriched and assembled on Ti_3_C_2_T_x_ nanosheet surfaces. As demonstrated by Cheng et al. [45] and Li et al. [46], neither external reductants nor surfactants were used in this experiment during the whole synthesis process. The effects of AuNPs on the enhancement of conductivity are illustrated by their combination with Ti_3_C_2_T_x_ nanosheets to form a conducting network structure. Increased active sites, ion transport, and electrolyte penetration are all facilitated by this arrangement.

### 3.2. Electrochemical Properties

#### 3.2.1. Three Electrode Setup

**Figure 5:**
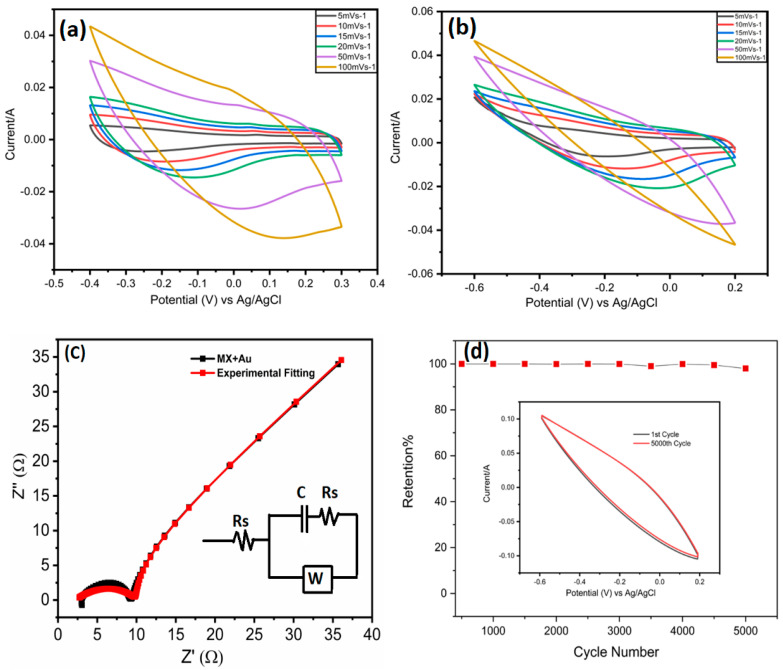
(**a**) CV curves of MXene at different scan rates in 3-electrode setup; (**b**) CV curves of MX/AuNPs in 1 M H_2_SO_4_ at different scan rates in 3-electrode setup; (**c**) Nyquist plots of MX/AuNPs, Inset shows the equivalent circuit model for the Nyquist plots; (**d**) MX/AuNPs electrode showing excellent cyclic stability with 98% capacitance retention at 100 mVs^−1^ 1M H_2_SO_4_ after 5000 cycles.

The free-standing MXene and MX/Au film were cut into circular electrodes with a diameter of about 1.2 cm, and the mass of each electrode was measured. The working electrodes in the three-electrode test were MXene and the MX/Au film, with platinum and Ag/AgCl electrodes serving as the counter and reference electrodes(as illustrated in Figure S1a). For the CV and GCD measurements, the voltage windows were 0.7 V and 0.8 V for MXene and MX/Au, respectively. Both films were subjected to EIS studies at perturbation voltage amplitudes of 5 mV and frequencies ranging from 10 MHz to 100 kHz. In addition, the CHI660E electrochemical workstation was used to achieve electrochemical performance. The CV profiles of MXene and MX/Au (Figure 5a,b) revealed that the valence state change of the titanium atoms and the pseudocapacitance owing to reversible redox reaction accounts for the majority of the capacitance.

The CV curves of pure MXene at various scan rates (5 mVs^−1^ to 100 mVs^−1^) in the voltage range of 0.3 to − 0.4 V (vs. Ag/AgCl) are shown in Figure 5a. At 5 mVs^−1^, MXene electrodes had a gravimetric capacitance of 383.5 Fg^−1^. The CV profiles did not show anodic or cathodic peaks, indicating that the MXene’s electrochemical charge storage process could be the dominating mechanism. However, as illustrated in Figure 5b, the addition of HAuCl_4_ significantly raised the MXene gravimetric capacitance by blocking MXene sheet restacking and assisting in the ion intercalation process. Therefore, the greater d-spacing can be attributed to the overall improvement in charge storage performance by minimizing the restacking that would cause reversible (de)intercalation.

The MX/Au electrode in three setups has been tested in 1 M H_2_SO_4_ and 3 M H_2_SO_4_ electrolytes (Figure 5b and Figure 7d). The MX/Au had shown a large gravimetric specific capacitance which is much higher than the pure MXene, confirming its high capacitance property. Likewise, MX/AuNPs composite electrode has a higher CV area than a pure MXene electrode due to the incorporation of AuNPs, demonstrating superior electrochemical capacitive performance. Furthermore, incorporating the AuNps boosted the performance of the voltage window. For instance, at a scan rate of 100 mVs^−1^, a decent specific capacitance of 131.9 Fg^−1^ was maintained (Figure 5b), demonstrating that the MX/Au has a sizably high-rate capability. The MX/Au acquired a series of massive specific capacitances of 468.36, 364.93, 324.53, 295.24, 177.03, and 131.93 Fg^−1^ in 1 M H_2_SO_4_ at scan speeds of 5, 10, 15, 20, 50, and 100 mVs^−1^ (Figure 5b). This pattern can be explained by the fact that the current responsiveness of all electrodes improves as the scan rate rises, which is the ideal behavior for a capacitor. Moreover, MX/AuNPs electrode showed excellent cyclic stability with 98% capacitance retention at 100 mVs^−1^ in 1 M H_2_SO_4_ after 5000 cycles (Figure 5d).

**Figure 6:**
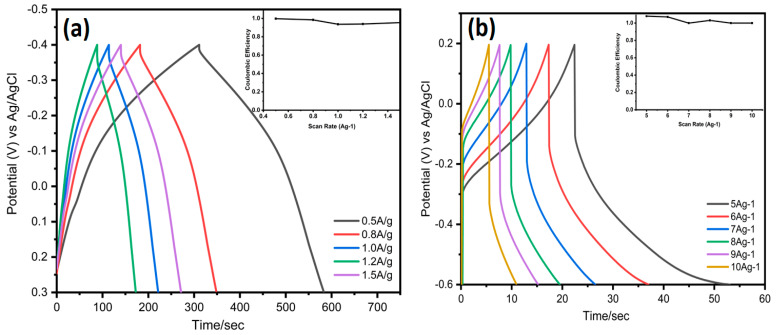

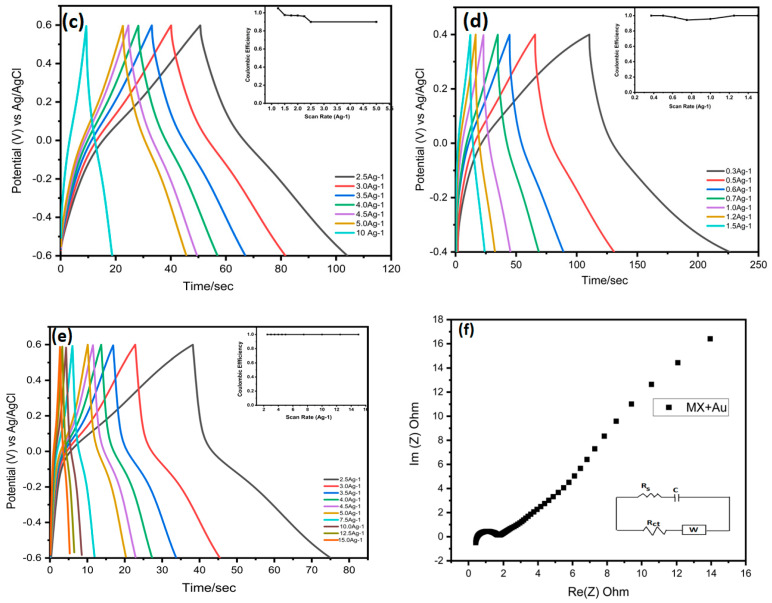
(**a**) GCD curves of MXene with different current densities, in 3-electrode setup, Inset shows the coulombic efficiency; (**b**) GCD curves of MX/AuNPs with different current densities, Inset shows the coulombic efficiency; (**c**) GCD curves of MXene Symmetric SC at different current densities, Inset shows the coulombic efficiency; (**d**) GCD curves of MX/AuNPs Symmetric SC at different current densities 1 M H_2_SO_4_, Inset shows the coulombic efficiency; (**e**) GCD curves of MX/AuNPs Symmetric SC at different current densities 3 M H_2_SO_4_, Inset shows the coulombic efficiency; (**f**) Nyquist plots of symmetric SC of MX/AuNPs and inset shows the equivalent circuit.

An increase in sweep rate limits the accessibility of the electrolyte ions in the electrode material. At quicker scan rates, only the electrode surface participates in the electrochemical process while the ions remain unable to enter inside the electrode material. These diffusion limits have less effect on the electrode, per CV curves. The galvanostatic charge and discharge curves for electrodes constructed of pure MXene and MXene/AuNps are shown in Figure 6a–e, respectively. The pseudocapacitive nature of all of them is demonstrated by a minor deviation from the ideal triangular shape, which is consistent with CV curve observations. The CV curves of a pure MXene electrode and MX/AuNps composite electrode were compared in Figure S6a at a scan rate of 5 mVs^−1^. Moreover, it can be seen that the CV of an integral area of MX/AuNps composite electrodes is significantly greater than that of a pure MXene electrode. The coulombic efficiency from GCD values for MXene and MXene AuNPs demonstrated the outstanding value and effective charge/discharge operation of MXene/AuNPs, as shown in the insets of Figure 6a,b. It should be highlighted that the AuNPs do not contribute in any capacitive way. Therefore, when HauCl_4_ solution is added in excess, the specific capacitance drops [47].

As shown in Figure 5c, the Nyquist plot of MX/AuNps consists of a quasi-semicircle in the high-frequency areas and a nearly vertical line in the low-frequency regions. In the high-frequency part, the semicircle arc shows the electrode surface properties and charge transfer resistance (Rct). Due to its greater conductivity for electronic application, improved ion and electrolyte transport, and bigger specific surface area for the active site, the pure MXene electrode has the lowest resistance value, Rct, based on the equivalent circuit fitting (Figure 5c inset).

#### 3.2.2. Two-Electrode Configuration

To assess the hybrid electrode’s feasibility in practical applications for flexible energy storage, the Swagelok cell design (as shown in Figure S1b) was utilized to build MXene and MX/AuNPs symmetric supercapacitors in 1 and 3 M H_2_SO_4_ aqueous electrolytes. In this system, two identical electrode pieces were used with a separator of cellulose membrane. Figure 6a–c depict the cyclic voltammetry graphs of the symmetric SCs at various scan rates. The 3 M H_2_SO_4_ MX/AuNPs composite electrodes performed the best among the three.

**Figure 7:**
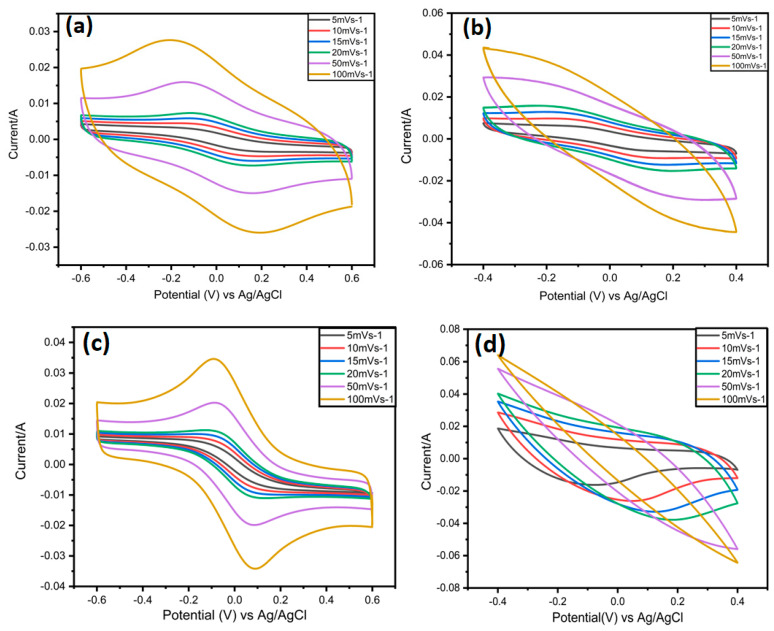
(**a**) CV curves of Symmetric SC of MXene at different scan rates 1 M H_2_SO_4_; (**b**) CV curves of Symmetric SC of MX/AuNPs at different scan rates in 1 M H_2_SO_4_; (**c**) CV curves of Symmetric SC of MX/AuNPs at different scan rates in 3 M H_2_SO_4_; (**d**) CV curves of MX/Au electrode in 3 M H_2_SO_4_.

In Figure 7c, CV curves of MX/AuNPs symmetric SCs in 3 M H_2_SO_4_ exhibit two redox peaks in the voltage range of 0.6 to −0.6 V at scan speeds ranging from 5 to 100 mVs^−1^, indicating that redox pseudocapacitance was dominance capacitance form. Even at 100 mVs^−1^, the morphology of the redox peaks is preserved with only a slight shift, indicating a high-rate capacity.

**Figure 8:**
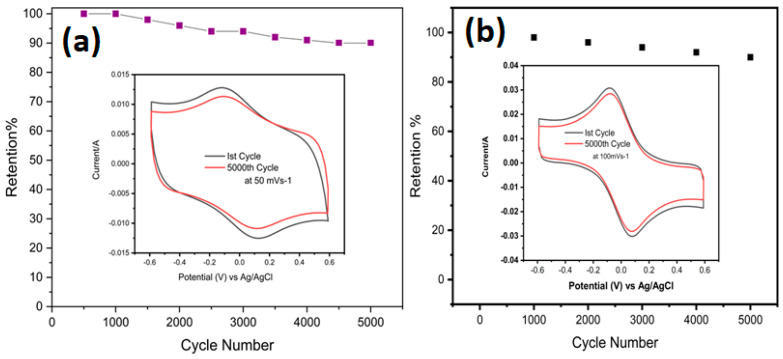
(**a**) Symmetric SC MXene showing good cyclic stability at 50 mVs^−1^ in 1 M H_2_SO_4_ after 5000 cycles; (**b**) Symmetric SC MX/AuNPs showing good cyclic stability at 100 mVs^−1^ in 3 M H_2_SO_4_ after 5000 cycles.

At lower scan rates, the CV curves for the Ti_3_C_2_T_x_ symmetric supercapacitor show two broader redox peaks (Figure 7a). With increasing scan rates, the redox peaks become less noticeable, especially during the discharging phase. This implies that the electrochemical processes of the symmetric MX/AuNPs supercapacitor and the symmetric MXene supercapacitor are different (Figure 7b). The CV curves of a pure MXene and MX/AuNps symmetric supercapacitor were compared in Figure S6b at a scan rate of 5 mVs^−1^.As expected, the symmetric SC device’s specific capacitance vs. scan rates exhibited enhanced capacitive behavior for the MX/AuNPs symmetric SC (Figure S11). The capacitance of the MX/AuNPs symmetric SC was 696.67 F g^−1^ at a scan rate of 5 mVs^−1^, in 3 M H_2_SO_4_, whereas the capacitance of the MXene symmetric supercapacitor was only 239 F g^−1^ (Figure 7a,c). Figure 8a,b demonstrates that our product has excellent cycling performance, retaining 90% of its capacitance after 5000 cycles. Symmetric SC MX and MX/Au cyclic stability performed at 50 mVs^−1^ and 100 mVs^−1^, respectively.

The high-frequency Nyquist curve (Figure S8) lacks a noticeable semi-circular shape indicating a lower Rct value of composite and superior electrical conductivity, while the MX/AuNPs show the semicircle (Figure 6f and Figure S7). The MX/AuNps symmetric supercapacitor shows a maximum energy density of 138.4 Wh kg^−1^ and a power density of 2076 W kg^−1^ which is much higher than that of the MXene symmetric SCs (47.5 Wh kg^−1^ energy density and 713 W kg^−1^ power density) and other previously reported MXene based symmetric supercapacitors as represented by the Ragone plots in Figure S12 and values represented by Table S2. As a result, it is expected that this simple method of integrating noble metal with Ti_3_C_2_T_x_ will improve overall electrochemical performance while maintaining electrode flexibility. It is also thought to be feasible for manufacturing high-energy-density flexible energy storage systems.

## 4. Conclusions

In summary, Ti_3_C_2_T_x_ nanosheets decorated with AuNPs were synthesized via a self-reduction procedure employing HAuCl_4_ aqueous solution. The combined solution was filtered through a vacuum to create the composite films. Then, MX/Au film was used as the electrode material in a supercapacitor operating in 3 M H_2_SO_4_. Compared to pure Ti_3_C_2_T_x_ electrodes and MX/AuNPs in 1 M H_2_SO_4_, the electrochemical properties of the MXene/AuNPs supercapacitor in 3 M H_2_SO_4_ were greatly improved. In contrast to prior reports, AuNps serve as the best intercalators in the hybrid structure, allowing the MXene active surface to be completely used. The resulting freestanding MX/AuNPs film supercapacitors displayed an outstanding performance like previously reported Ti_3_C_2_T_x_-based symmetric SCs, and exhibited an ultrahigh specific capacitance of 697 Fg^−1^, which is almost twice that of the Ti_3_C_2_T_x_. Additionally, it demonstrates exceptional flexibility as displayed by being bent, coiled, and even folded without cracking. Compared to Ti_3_C_2_T_x_ symmetric supercapacitors, the proposed symmetric supercapacitor device has excellent energy density (139 Wh kg^−1^) and power density (2076 W kg^−1^). We anticipate that this research will speed up the development of MXene-based flexible electrodes into high-energy-density energy storage systems.

## Data Availability

The data presented in this study are available within the article.

## Supplementary Materials

The following supporting information are available online at https://www.mdpi.com/article/10.3390/nano12193294/s1, Figure S1: (a) Illustration of three-electrode setup; (b) Digital photo of two setup using Swagelok cell, Figure S2: (a) the digital photograph of the free-standing film, showing excellent flexibility at different deformation statuses, which can be bent, rolled, and even folded; (b) digital photo along the scale., Figure S3: (a) EDS spectrum of MX/AuNP film; (b) The elemental mapping of MX/Au free-standing film. Figure S4: (a) EDS spectrum of MXene film; (b) The elemental mapping of MXene free-standing film., Figure S5: (a, b) SEM images of Mxene films; (c)TEM image of Mxene film (d) HRTEM image of Mxene film. Figure S6: (a) CV curves of MX and MX/AuNPs in three electrodes setup at 5mVs^−1^; (b) CV curves Symmetric SCs of MXene and MX/AuNPs at 5mVs^−1^. Figure S7: Nyquist plots of symmetric SC of MX/AuNPs, Inset shows the equivalent circuit model Figure S8: Nyquist plots of symmetric SC of Mxene. Figure S9: The survey spectra of Mxene film Figure S10: Graph between the peak current densities of all the tested electrodes Vs square root of Scan Rate. Figure S11: Specific capacitance of MXene and MX/AuNPs electrode and Symmetric SCs vs. the scan rate., Figure S12: Ragone plots displaying energy and power densities of MX/AuNPs symmetric supercapacitor in comparison with other MXene-based supercapacitors, Table S1:EDS results of the Mxene and MX/AuNPs, Table S2: Comparison of electrochemical performance of different MXene-based materials as symmetric supercapacitor. References [32,42,48,49,50] are cited in the Supplementary Materials.
