# Supplementary material for: Ultrahigh Energy and Power Densities of d-MXene-Based Symmetric Supercapacitors
Source: Nanomaterials (Basel). 2022 Sep 22;12(19):3294. doi: 10.3390/nano12193294 (PMC9565486; doi:10.3390/nano12193294)
Supplement: Supplementary file 1 [file nanomaterials-12-03294-s001.zip › nanomaterials-1894121-supplementary.pdf]

nanomaterials

Supplementary Information

Ultrahigh Energy and Power Densities of d-MXene-Based Symmetric Supercapacitors

Beenish Mustafa [1], Wengang Lu [1], Zhiyuan Wang [1], Fuzhuo Lian [1], Andy Shen [2], Bing Yang [2], Jun Yuan [2], Chang Wu [2], Yangbowen Liu[2], Weiwei Hu [3], Lei Wang [1,4,*] and Geliang Yu [1,4,*]

[1] National Laboratory of Solid-State Microstructures, School of Physics, Nanjing University, No. 22, Hankou Road, Nanjing 210093, China; dg1822501@smail.nju.edu.cn (B.M.); mf1922006@smail.nju.edu.cn (W.L.); mg21220104@smail.nju.edu.cn (Z.W.); mg20220101@smail.nju.edu.cn (F.L.)
[2] Hubei Jiufengshan Laboratory, Wuhan 430206, China; andyshen@jfslab.com.cn (A.S.); yangbing@jfslab.com.cn (B.Y.); yuanjun@jfslab.com.cn (J.Y.); wuchang@jfslab.com.cn (C.W.); liuyangbowen@jfslab.com.cn (Y.L.)
[3] Jiangsu Industrial Technology Research Institute, Nanjing 210093, China; huww@jitri.cn
[4] Collaborative Innovation Centre of Advanced Microstructures, Nanjing University, Nanjing 210093, China
* Correspondence: leiwang@nju.edu.cn (L.W.); yugeliang@nju.edu.cn (G.Y.)

Citation: Mustafa, B.; Lu, W.; Wang, Z.; Lian, F.; Shen, A.; Yang, B.; Yuan, J.; Wu, C.; Liu, Y.; Hu, W.; et al. Ultrahigh Energy and Power Densities of d-MXene-Based Symmetric Supercapacitors. *Nanomaterials* **2022**, *12*, 3294. https://doi.org/10.3390/nano12193294

Academic Editor: Sergio Brutti

Received: 16 August 2022
Accepted: 14 September 2022
Published: 22 September 2022

Publisher's Note: MDPI stays neutral with regard to jurisdictional claims in published maps and institutional affiliations.

Copyright: © 2022 by the authors. Licensee MDPI, Basel, Switzerland. This article is an open access article distributed under the terms and conditions of the Creative Commons Attribution (CC BY) license (https://creativecommons.org/licenses/by/4.0/).

Electrochemical Experiment and measurement:

All electrochemical tests were conducted by using an Electrochemical workstation CHI660E by using two types of setup, a three-electrode setup, and two electrodes as shown in Fig. S1a and S1b. In the three-electrode setup, a platinum electrode was used as the counter electrode, both Mxene and MX/AuNPs free-standing films are used as a working electrode and Ag/AgCl was the reference electrode. Aqueous 1M and 3 M H_2SO_4 electrolyte and a cellulose membrane was used as the separators. All the electrodes were pre-cycled for 20 cycles using Cyclic Voltammetry (CV) at 20 mV s^{-1} before the actual electrochemical tests were performed. Moreover, the electrodes were dipped in the aqueous solution H_2SO_4 for 24hrs before testing. The CV tests for different MXene electrodes were performed at scan rates of 5-100 mV s^{-1}. Galvanostatic charge-discharge (GCD) cycling was performed at different current densities. To check the cyclic stability and capacitance retention CV cycle was performed at 50 and 100 mVs^{-1} for 5000cycles. The specific capacitance was calculated from the CV curves in the three-electrode setup can be calculated using the following formula.

$$C_s = \frac{\int I \, dV}{m\nu\Delta V} \tag{S1}$$

Where, ΔV (V) is the potential window, m (g) is the mass of the working electrode, V (V) is the potential versus reference electrode, I (A) is the response current, ν (V s^{-1}) is the scan rate, Cs (F g^{-1}) is specific capacitance.

In the two-electrode cell, Cs values of the electrodes in the symmetric supercapacitor can be obtained by the following equation using the CV curves.

$$C_s = \frac{\int I \, dV}{M\nu\Delta V} \tag{S2}$$

where M (g) is the mass of both electrodes and ν (V s^{-1}) is the scan rate. The volumetric capacitance of film electrodes can be calculated according to Eqs. 3 and 4:

$$C_v = \rho C_s \tag{S3}$$

$$\rho = \frac{m}{sd} \tag{S4}$$

where S (cm^{-2}) and d (cm) are the surface area and thickness of film electrode, respectively.

The Energy density (Wh kg^{-1}) and power density (W Kg^{-1}) can be calculated by using following equations.

$$E = \frac{0.5 \times C_S \times (\Delta V)^2}{3.6} \quad (S5)$$

$$p = \frac{E \times 3600}{\Delta t} \quad (S6)$$

where Δt is the discharge time according to the CV curves ($\Delta t = \frac{\Delta V}{\nu}$)

The coulombic efficiency was calculated from GCD curves according to the following equation:

$$c_E = \frac{\Delta t_d}{\Delta t_c} \quad (S7)$$

Where, Δt_d and Δt_c are the time of discharging and charging, respectively, and CE is coulombic efficiency.

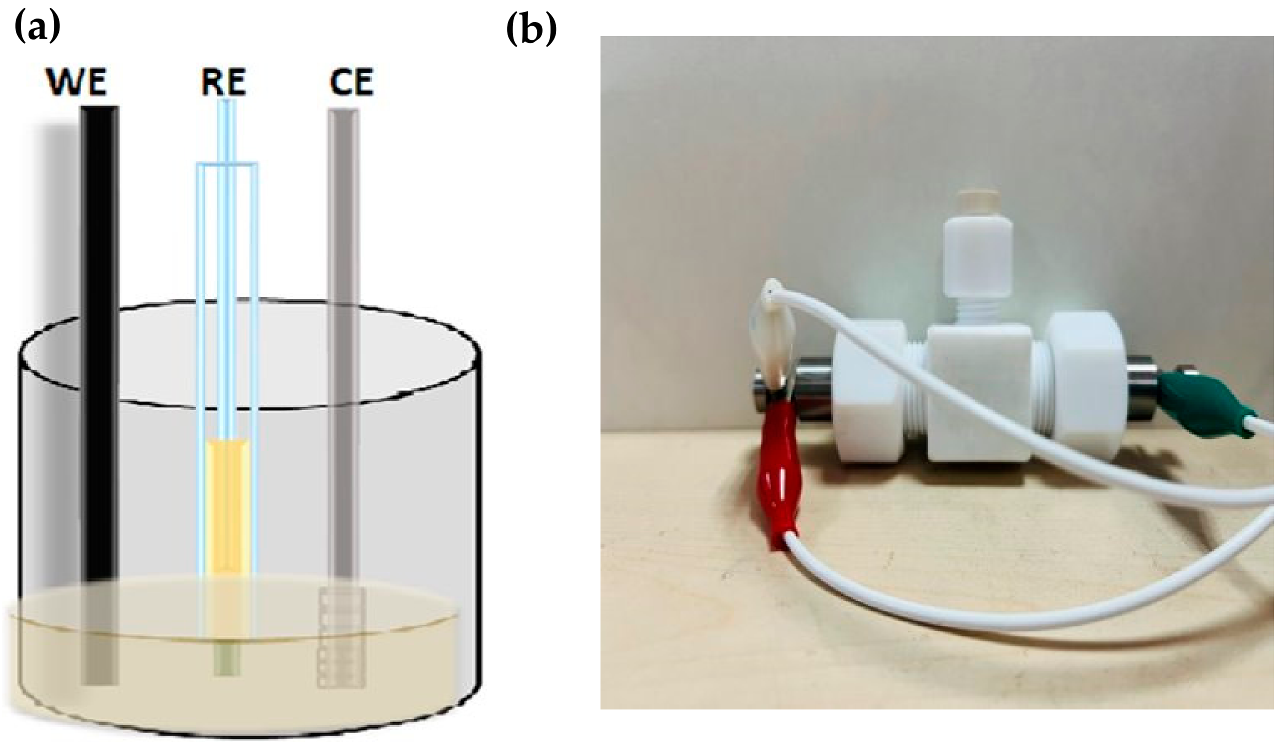

Figure S1. (a) Illustration of three-electrode setup; (b) Digital photo of two setup using Swagelok cell.

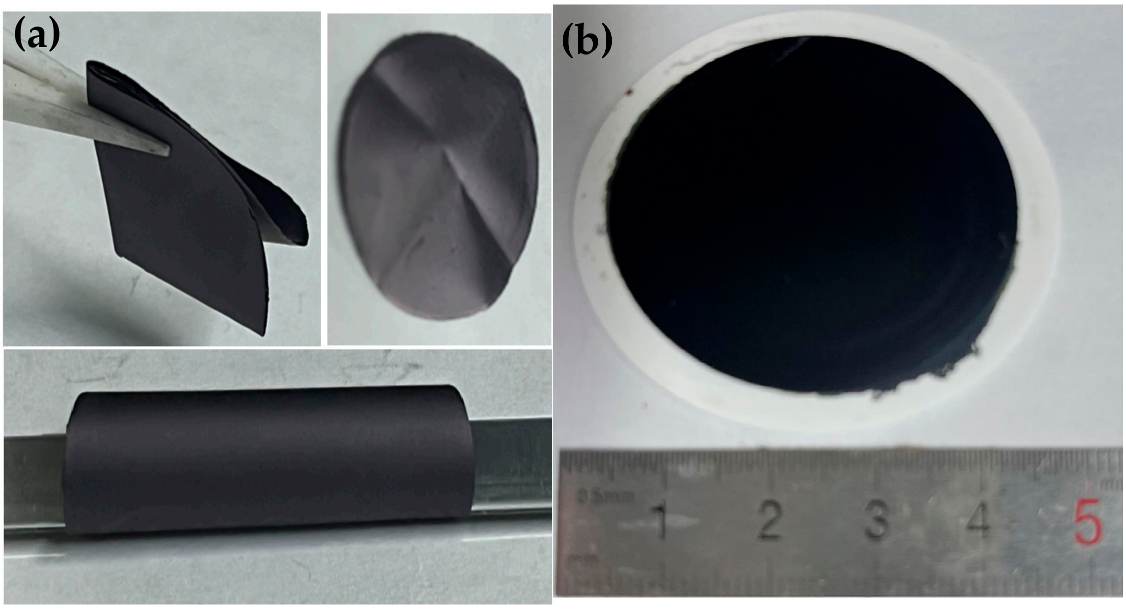

Figure S2. (a) the digital photograph of the free-standing film, showing excellent flexibility at different deformation statuses, which can be bent, rolled, and even folded; (b) digital photo along the scale.

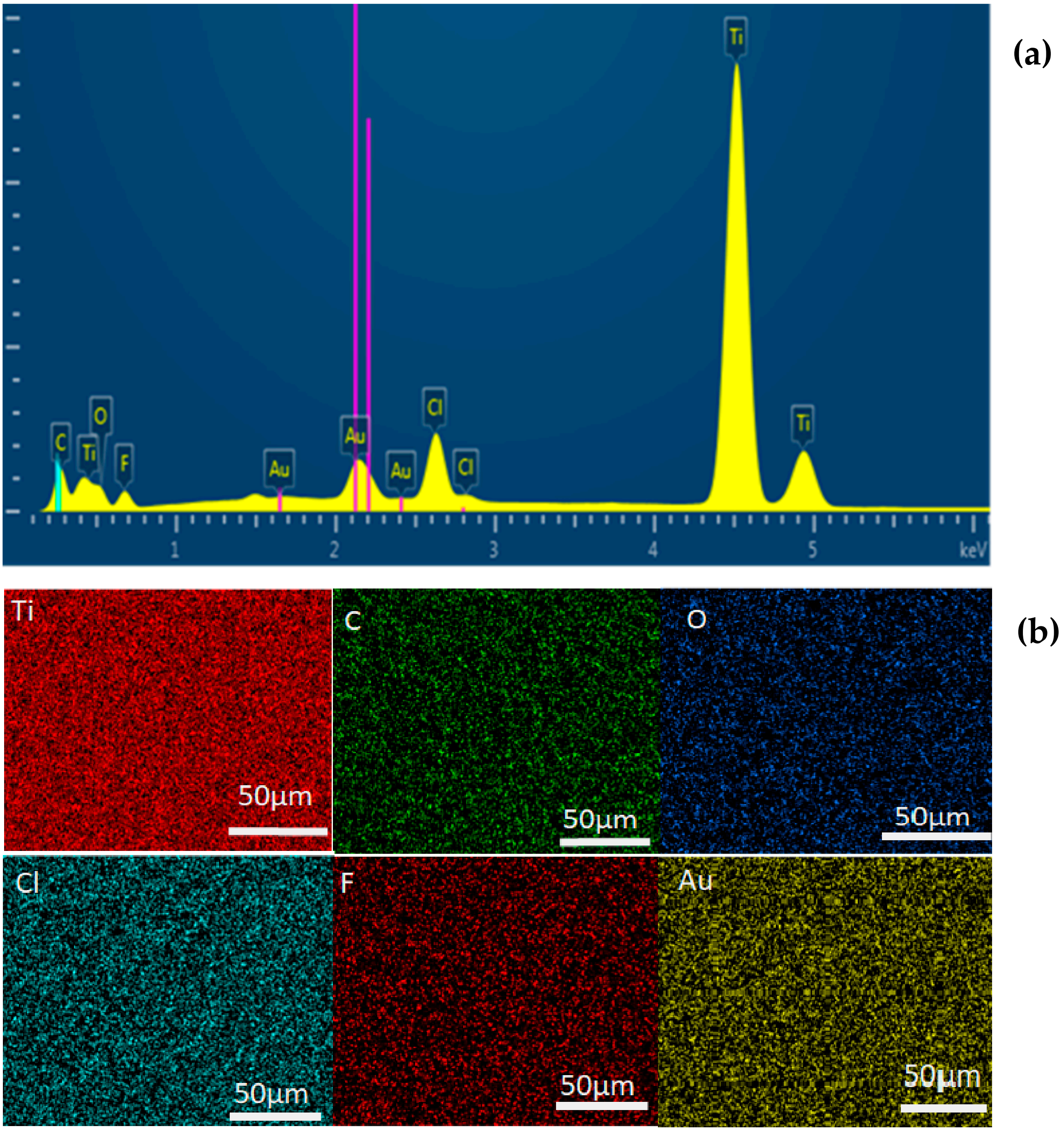

Figure S3. (a) EDS spectrum of MX/AuNP film; (b) The elemental mapping of MX/Au free-standing film.

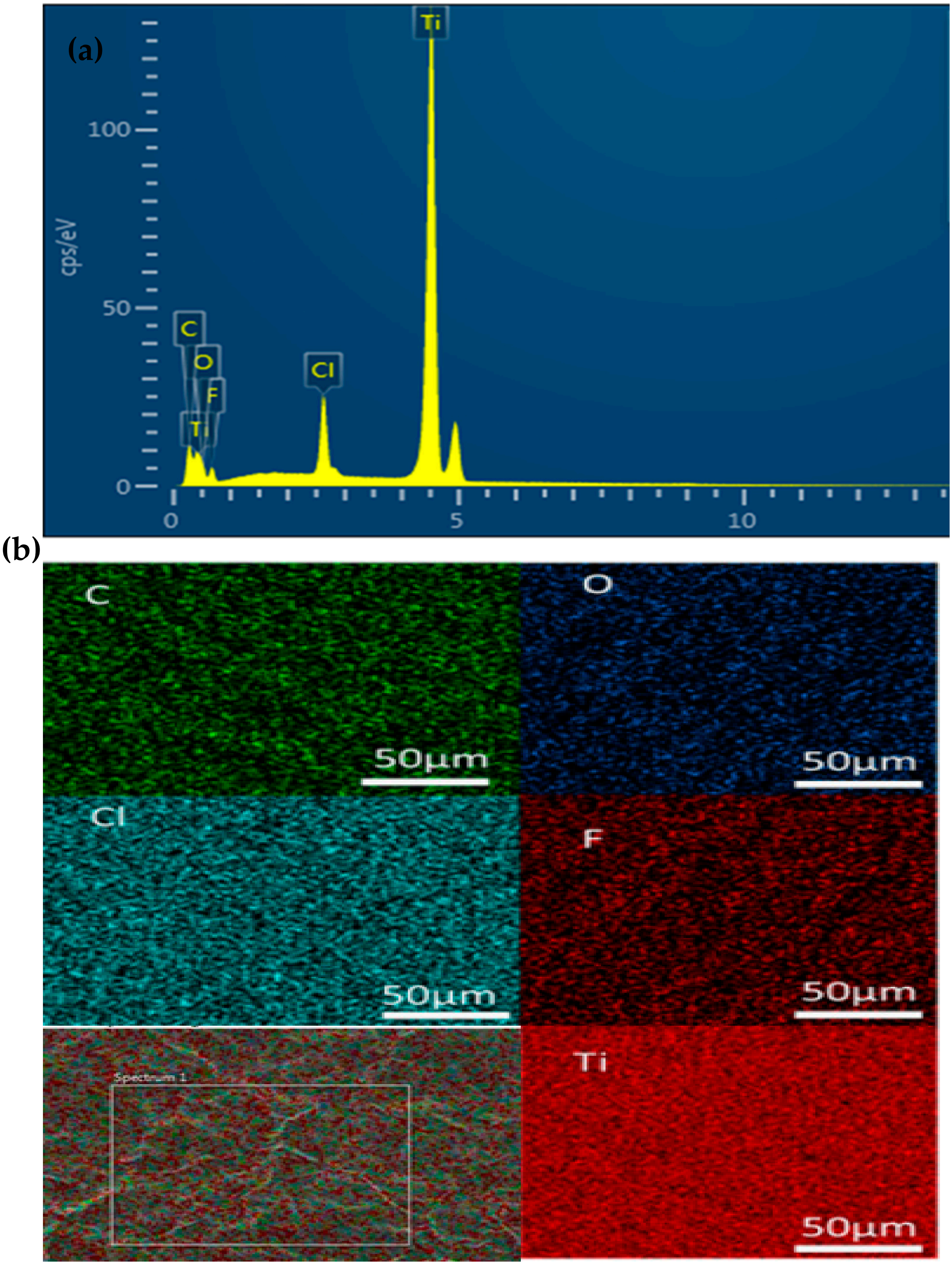

Figure S4. (a) EDS spectrum of MXene film; (b) The elemental mapping of MXene free-standing film.

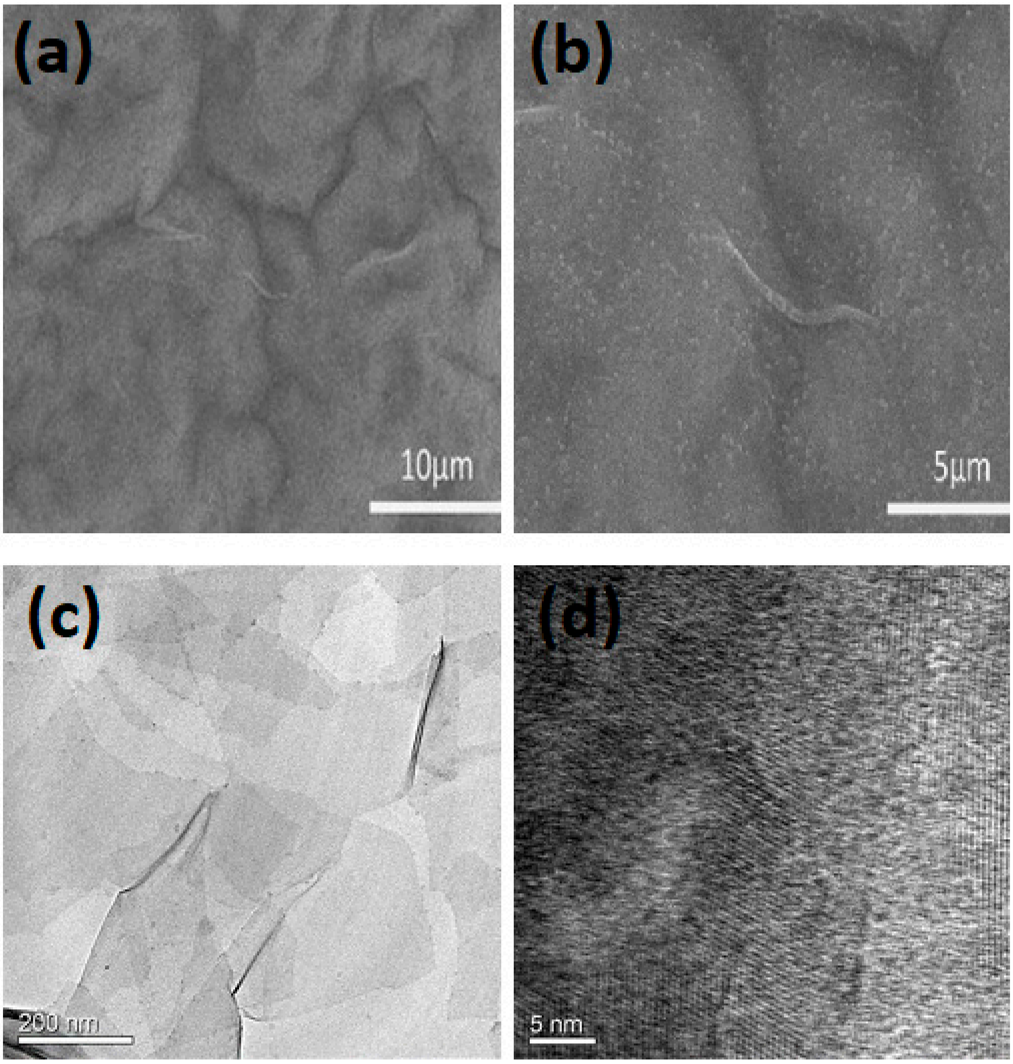

Figure S5. (a, b) SEM images of Mxene films; (c)TEM image of Mxene film (d) HRTEM image of Mxene film.

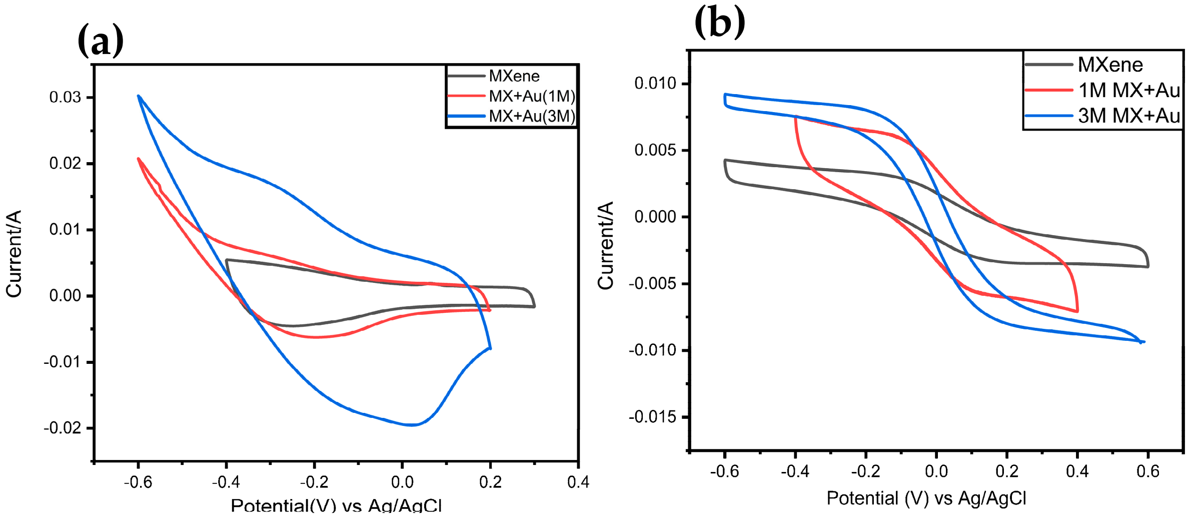

Figure S6. (a) CV curves of MX and MX/AuNPs in three electrodes setup at 5mVs $^{-1}$; (b) CV curves Symmetric SCs of MXene and MX/AuNPs at 5mVs $^{-1}$.

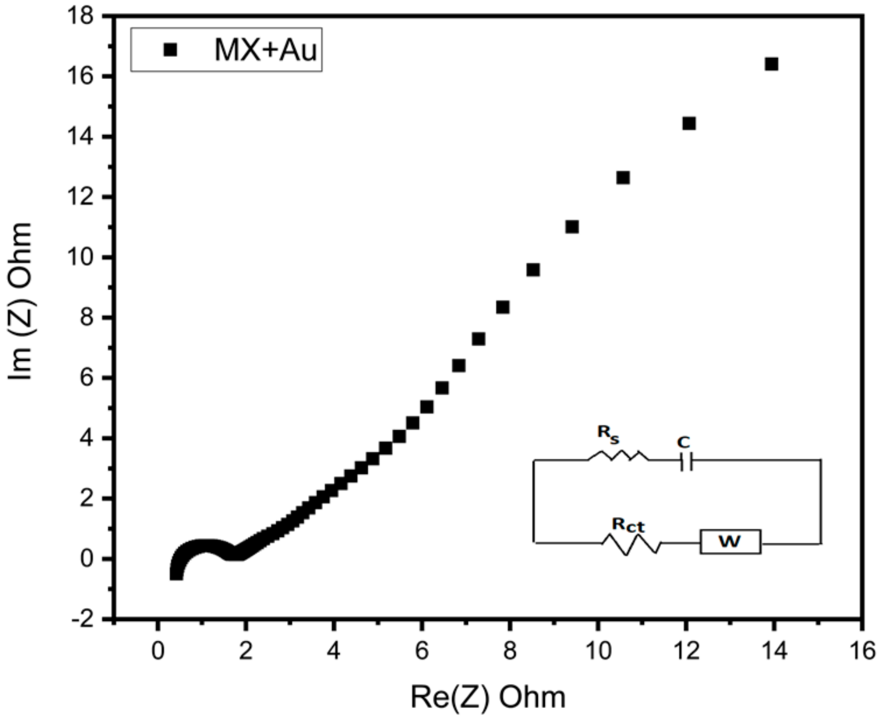

Figure S7. Nyquist plots of symmetric SC of MX/AuNPs, Inset shows the equivalent circuit model.

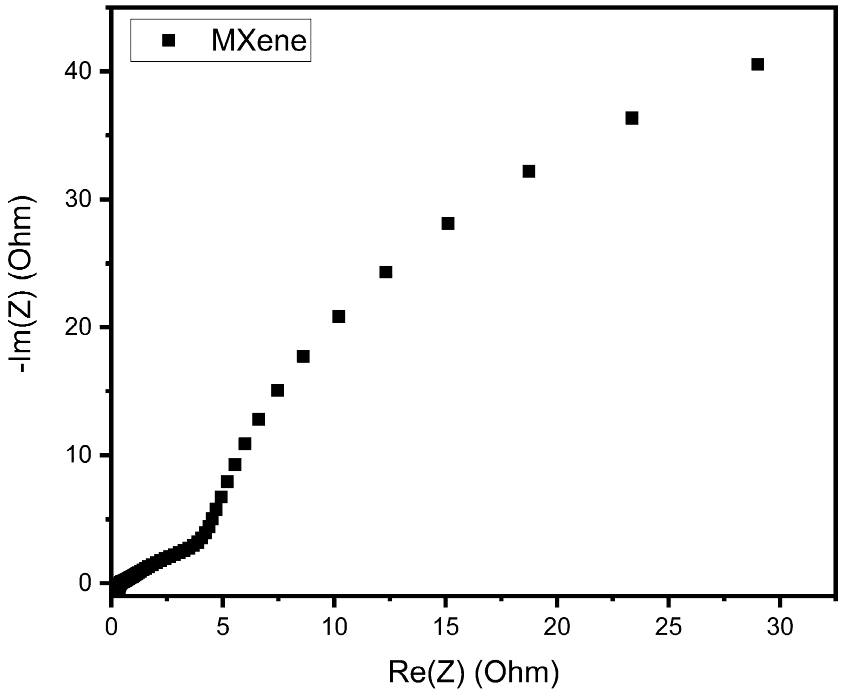

Figure S8. Nyquist plots of symmetric SC of Mxene.

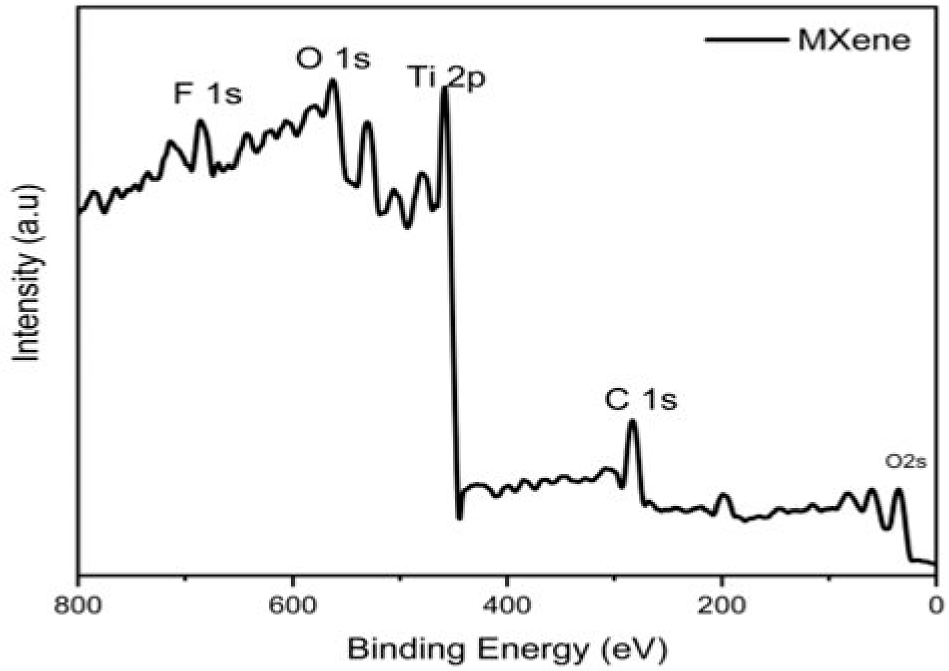

Figure S9. The survey spectra of Mxene film.

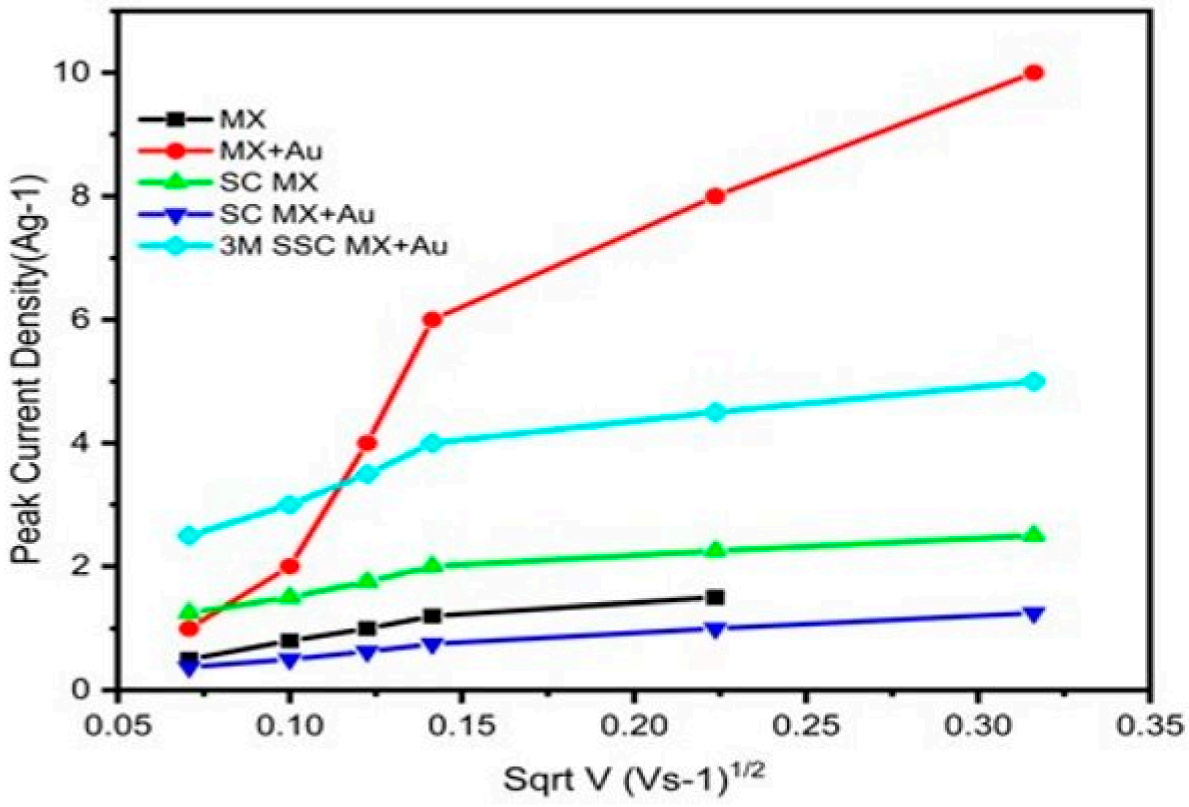

Figure S10. Graph between the peak current densities of all the tested electrodes Vs square root of Scan Rate.

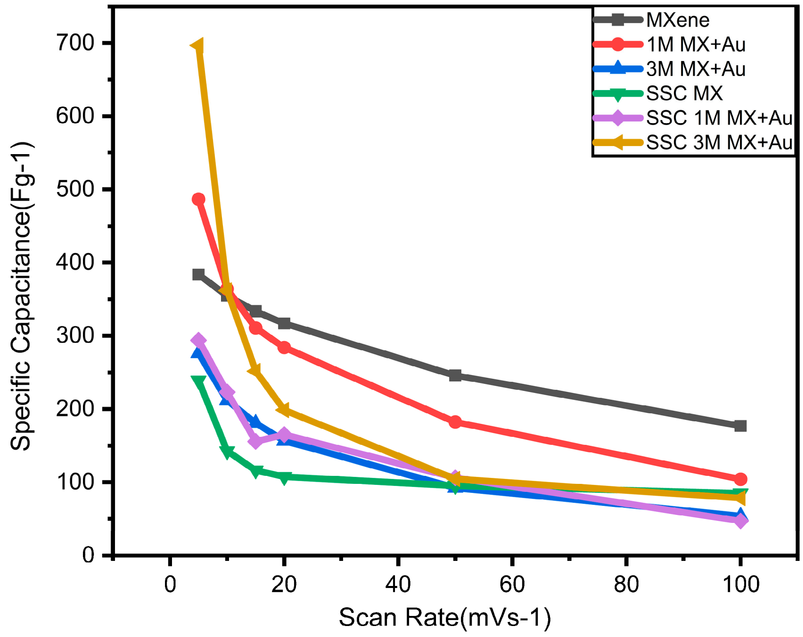

Figure S11. Specific capacitance of MXene and MX/AuNPs electrode and Symmetric SCs vs. the scan rate.

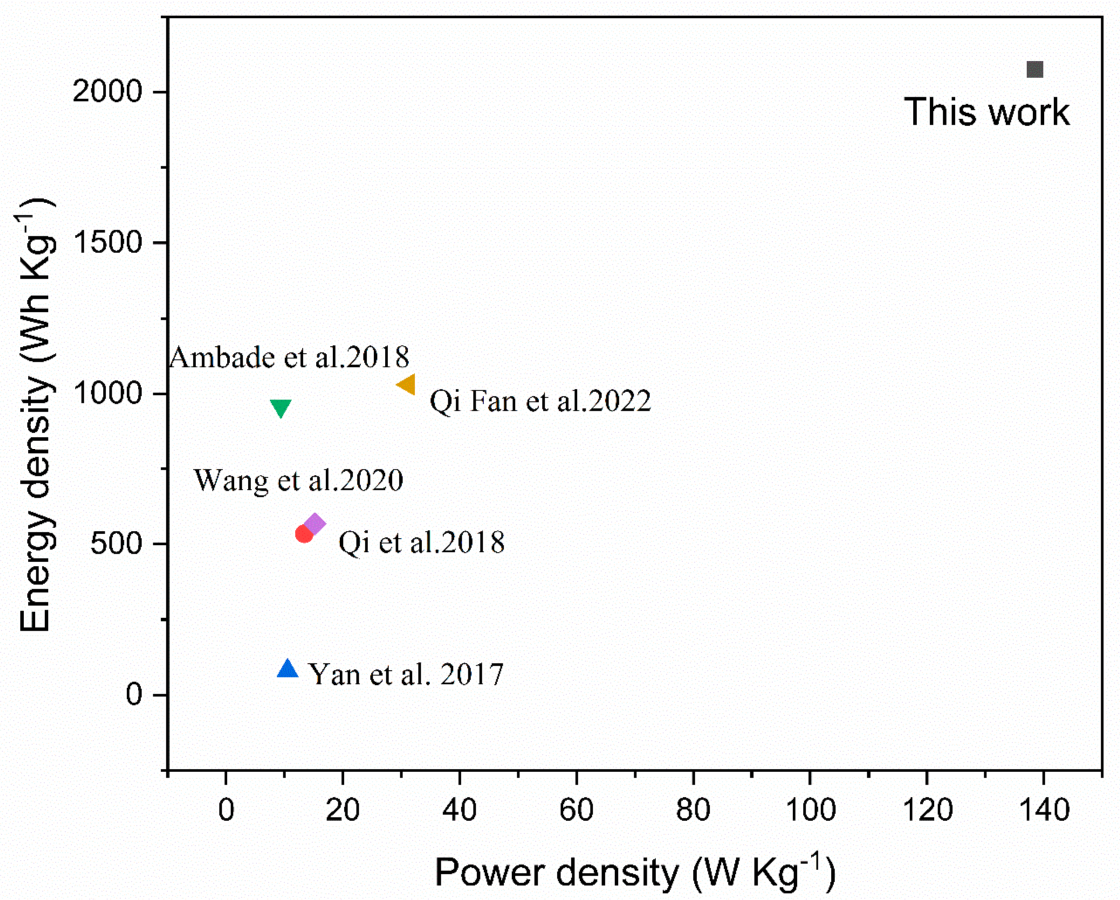

Figure S12. Ragone plots displaying energy and power densities of MX/AuNPs symmetric supercapacitor in comparison with other MXene-based supercapacitors.

Table S1. EDS results of the Mxene and MX/AuNPs.

Element	MXene		MX/AuNps	
	Atomic %	Weight %	Atomic %	Weight %
Ti	36.18	62.61	32.92	32.92
C	26.04	11.30	28.42	28.42
O	19.77	11.43	18.36	18.36
F	14.16	9.72	15.84	15.84
Cl	3.86	4.94	3.48	3.48
Au	-	-	0.98	6.80

Table S2. Comparison of electrochemical performance of different MXene-based materials as a symmetric supercapacitor.

Electrode material	Electrolyte	Specific Capacitance (Fg $^{-1}$)	Energy Density (Wh Kg $^{-1}$)	Power Density (W kg^{-1})	Ref.
MX/AuNP	3M H_2SO_4	696.67	138.44	2076	This work
M/ MoO_3	1M H_2SO_4	545	13.4	534.6	1
M/G-5%	3M H_2SO_4	335	10.5	80.3	2

MXene/WO_3	0.5M H_2SO_4	566	9.32	960	3
TCBOC	1 M KOH	247.8	15.2	567.4	4
PPy-MXene	Ionic Liquid	51.85	31.2	1030.4	5

References

1. Wang, Y.; Wang, X.; Li, X.; Liu, R.; Bai, Y.; Xiao, H.; Liu, Y.; Yuan, G., Intercalating ultrathin moo3 nanobelts into mxene film with ultrahigh volumetric capacitance and excellent deformation for high-energy-density devices. *Nano-micro letters* **2020**, *12*, 1-14.
2. Yan, J.; Ren, C.E.; Maleski, K.; Hatter, C.B.; Anasori, B.; Urbankowski, P.; Sarycheva, A.; Gogotsi, Y., Flexible mxene/graphene films for ultrafast supercapacitors with outstanding volumetric capacitance. *Advanced Functional Materials* **2017**, *27*, 1701264.
3. Ambade, S.B.; Ambade, R.B.; Eom, W.; Noh, S.H.; Kim, S.H.; Han, T.H., 2d ti3c2 mxene/wo3 hybrid architectures for high-rate supercapacitors. Advanced Materials Interfaces 2018, 5, 1801361.
4. Xia, Q.X.; Shinde, N.M.; Yun, J.M.; Zhang, T.; Mane, R.S.; Mathur, S.; Kim, K.H., Bismuth oxychloride/mxene symmetric supercapacitor with high volumetric energy density. Electrochimica Acta 2018, 271, 351-360.
5. Fan, Q.; Zhao, R.; Yi, M.; Qi, P.; Chai, C.; Ying, H.; Hao, J., Ti3c2-mxene composite films functionalized with polypyrrole and ionic liquid-based microemulsion particles for supercapacitor applications. Chemical Engineering Journal 2022, 428, 131107.
